# A Mechanism for Chronic Filarial Hydrocele with Implications for Its Surgical Repair

**DOI:** 10.1371/journal.pntd.0000695

**Published:** 2010-06-01

**Authors:** Joaquim Norões, Gerusa Dreyer

**Affiliations:** 1 Departamento de Cirurgia, Centro de Ciências da Saúde, Universidade Federal de Pernambuco, Recife, Pernambuco, Brazil; 2 Núcleo de Ensino, Pesquisa e Assistência em Filariose (NEPAF), Hospital das Clínicas, Universidade Federal de Pernambuco, Recife, Pernambuco, Brazil; 3 Organização Não governamental Amaury Coutinho para Doenças Endêmicas e Tropicais, Recife, Pernambuco, Brazil; 4 Centro de Pesquisas Aggeu Magalhães, Fundação Oswaldo Cruz (FIOCRUZ), Recife, Pernambuco, Brazil; Ghana Health Service, Ghana

## Abstract

**Background:**

Chronic hydrocele is the most common manifestation of bancroftian filariasis, an endemic disease in 80 countries. In a prospective study, we evaluated the occurrence of intrascrotal lymphangiectasia, gross appearance/consistency of the testis, and the efficacy of complete excision of hydrocele sac in patients living in a bancroftian filariasis endemic area who underwent hydrocelectomy at the Center for Teaching, Research and Tertiary Referral for Bancroftian Filariasis (NEPAF).

**Methodology/Principal Findings:**

A total of 968 patients with uni- or bilateral filarial hydrocele (Group-1) and a Comparison Group (CG) of 218 patients from the same area who already had undergone hydrocele-sac-sparing hydrocelectomy elsewhere were enrolled at NEPAF. Twenty-eight patients from the Comparison Group with hydrocele recurrence were re-operated on at NEPAF and constitute Group-2. In Group-1 a total of 1,128 hydrocelectomies were performed (mean patient age of 30.3yr and mean follow-up of 8.6yr [range 5.3–12]). The hydrocele recurrence rates in Group-1 and in the Comparison Group (mean age of 31.5 yr) were 0.3%, and 19.3%, respectively (p<0,001). There was no hydrocele recurrence in Group-2 (mean patient age of 25.1yr and mean follow-up of 6yr [range 5–6.9]). Per surgically leaking or leak-prone dilated lymphatic vessels were seen in the inner or outer surface of the hydrocele sac wall or in surrounding tissue, particularly in the retrotesticular area, in 30.9% and in 46.3% of patients in Group-1 and Group-2, respectively (p = 0.081). The testicles were abnormal in shape, volume, and consistency in 203/1,128 (18%) and 10/28 (35.7%) of patients from Group-1 and Group-2, respectively (p = 0,025).

**Conclusions/Significance:**

Lymph fluid from ruptured dilated lymphatic vessels is an important component of chronic filarial hydrocele fluid that threatens the integrity of the testis in an adult population living in bancroftian filariasis endemic areas. To avoid hydrocele recurrence the authors advise complete excision of hydrocele sac and when identified, leaking or leak-prone dilated lymphatic vessels should be sutured or excised.

## Author Summary

Chronic hydrocele is the accumulation of fluid around the testis leading to an increase in the volume of the scrotal contents. Depending on the volume of fluid, hydrocele can be disfiguring and even incapacitating. Chronic hydrocele has multiple etiologies, but irrespective of the cause, surgery is the standard form of treatment and this can be done using different surgical techniques. The prevalence of chronic hydrocele in bancroftian filariasis endemic areas—a parasitic disease transmitted by mosquito—is very high and represents the most common clinical manifestation of bancroftosis, following by swollen legs of lower limbs or lymphedema among women. In Greater Recife, northeastern, Brazil, a bancroftian filariasis endemic area, a pioneering, prospective surgical study proposes a new mechanism for filarial-induced hydrocele and presents evidence that the filarial hydrocele fluid may damage the testis. Thus, based on the findings presented, the authors propose that in bancroftian filariasis endemic areas hydrocele patients should be operated on using a specific surgical technique in order to avoid recurrence of the disease, and consequently, additional damage to the testicle.

## Introduction

Bancroftian filariasis is a mosquito-borne parasitic disease that affects approximately 100 million persons worldwide. It is estimated that 40 million persons suffer from the chronic disfiguring manifestations of this disease, including 27 million men with testicular hydrocele, lymph scrotum, or elephantiasis of the scrotum. The genital pathology caused by bancroftosis is impressively debilitating and economically punishing for huge numbers of adult males throughout endemic countries [1].

There is consensus that hydrocele is the most frequent clinical manifestation of bancroftian filariasis [2]. On the other hand, few systematic studies of the prevalence or incidence of hydrocele in temperate climates have been published. In spite of this, there appears to be a difference between the hydrocele prevalence in temperate countries and tropical and subtropical ones. In the tropics, hydrocele is a very frequent condition [3]. In locations where bancroftian filariasis is highly endemic, up to 40% of adult males are reported to have testicular hydrocele [4], [5].

With exception of its posterior aspect, the testicle is covered by the tunica vaginalis formed by two layers. The visceral layer and parietal layer are in direct contact with the testis and the scrotum wall, respectively. Both layers have secretory ability and only the parietal layer has resorption property. Between the layers there is a space called vaginal cavity. The balance of secretory and absorptive functions of these layers results in a small volume (0.5 to 2.0 mL) of straw colored fluid in this cavity. Outside of congenital hydrocele, the general mechanism accepted for abnormal chronic accumulation of fluid in the vaginal cavity, known as acquired hydrocele, irrespectively of the etiology, is unbalanced process between fluid production by the mesothelial cells of the inner surface of the tunica vaginalis and fluid absorption by the draining lymphatic vessels of the parietal layer. Surgery using different techniques is the standard treatment form for chronic hydrocele, regardless of the etiology, and the rationale for its repair is to expose, permanently, the secretory surface of tunica vaginalis to the absorbing surface of scrotal wall.

In Greater Recife, northeastern, Brazil, a bancroftian filariasis endemic area [6] the prevalence of acquired hydrocele in the adult male population is high and hydrocelectomy represents a commonly performed procedure in public hospitals. At these hospitals, the surgeons' preference is for surgical techniques in which the hydrocele sac is opened, everted with or without partial resection of the sac, and the edges sutured behind the testis (J. Norões, personal communication).

The purpose of this study is to evaluate the occurrence of lymphangiectasia in scrotal contents, the morphology and consistency of the testicles and, hydrocele recurrence using the complete excision of tunica vaginalis in hydrocele patients living in a bancroftian filariasis endemic area.

## Methods

This study was carried out between March 1994 and March 2006 as part of a larger comprehensive study on different aspects of urological manifestation of bancroftian filariasis based on clinical, parasitological, chemotherapeutical, ultrasonographic, biochemical, surgical and histopathological observations [7]–[16]. The study was approved by the Ethics Committee of Hospital das Clinicas at Federal University – Pernambuco, Brazil. It comprised three groups.

### Group-1 (G1)

Patients who underwent their first operation at NEPAF. Patients were selected using the following criteria: (a) aged between 18 and 40 year-old at the time of surgery and had signed the inform consent forms, which included permission to use his medical information for scientific publication; (b) documented uni- or bilateral hydrocele by physical examination (inspection and bimanual palpation) and ultrasonography of the scrotal area; (c) currently live or have lived in a house with at least one person with documented *W. bancrofti* microfilaremia: (d) do not have ipsilateral inguinal hernia; (e) no history of past ipsilateral inguinal hernia surgical repair, urological surgery, or intra-scrotal inflammation from testicular trauma or bacterial infection; (g) no current evidence of intrascrotal neoplasia; (g) no current ultrasonographic evidence of severe testicular damage which could anticipate the need of orchiectomy during hydrocelectomy; (h) demonstrated straw-colored hydrocele fluid during hydrocelectomy; (i) have at least five years of postoperative follow up; (j) have lived in a filariasis-endemic area for their entire life; (k) no medical contraindication for hydrocelectomy. Eligible patients were operated on by the same surgeon.

### Comparison Group (CG)

Between January 1999 and March 2001 any patient with bancroftian filariasis active infection/disease referred to NEPAF underwent a family protocol investigation that included the collection of information about current or past filarial infection/disease in family members of the patient. One of the questions to address hydrocele occurrence was whether or not hydrocelectomy had been performed in any family member. The family member(s) identified for previous hydrocelectomy was/were invited to NEPAF and selected according the following inclusion criteria: (1) if they lived in a house where at least one person had been positive for *Wuchereria bancrofti* microfilaria in the blood, (2) if they had had hydrocelectomy performed between the ages of 18 and 40 at public hospitals in Greater Recife and, (3) if they have signed the inform consent form. For each individual, information regarding the hydrocelectomy technique used was retrospectively gathered from the patient's medical records. All included, consenting individuals underwent physical and ultrasound examinations of the scrotal area.

### Group-2 (G2)

Patients from CG who underwent hydrocelectomy at NEPAF for hydrocele recurrence. Inclusion criteria were the same used for G1 except that the previous hydrocelectomy was performed elsewhere.

Hydrocelectomies were performed under NEPAF protocol, which included total excision of tunica vaginalis parietal, regional anesthesia and prophylactic antibiotic therapy. A povidone-iodine scrub was used for preoperative skin preparation. After the surgical area was prepared and draped an incision was made parallel to the median raphe and deepened in the layers of the scrotum wall. The dissection of the hydrocele sac was made until it was completely separated from its surrounding tissue. After meticulous hemostasis, the hydrocele fluid was drained completely from the sac using a 16–18 gauge intravenous catheter and a syringe. Only in patients presenting testes abnormalities (see below) was the volume considered in the analysis. In patients presenting with two hydrocele sacs, the hydrocele volume was considered the sum of the volume of both sacs. Between two clamps, a middle line incision was made in the anterior aspect of the sac wall in its midpoint along its vertical axis and extended, cephalically, until the limit of the cord close to the head of the epididymis and, caudally, until the proximity of its tail. After the hydrocele sac had been opened it was excised all around, close to its reflexion onto the visceral layer. Pre and post hydrocele sac excision a careful examination was done to look for dilated lymphatic vessels, whether leaking or not, especially in the vicinity of the excised sac wall. After identification, all visible dilated lymphatic vessels were sutured or ligated and excised. In thin-walled hydrocele sac any visible bleeding at the incised site was coagulated. In thick-walled cases the cutting margin was marsupialized by suturing it with a 4-0 chromic catgut continuous interlocking suture. Once hemostasis was achieved, the testis was returned to the scrotum and the wound was closed in two layers. The inner layer was closed with an interrupted 4-zero chromic catgut suture and the skin and dartos with a continuous 3-0 monofilament nylon suture. As a rule, no drains were used. A small dressing and a slight compressing scrotal bandaging were then applied. In cases with bilateral hydrocele, the same procedure was performed on the contra-lateral side using the same surgical procedure. The post-operative management included oral analgesic if necessary, scrotal support and, “ice bag” four times a day during the hospitalization period. At discharge the patients were instructed to perform personal hygiene twice a day with soap and clean water with changing of underwear. Underwear and soap were provided by NEPAF when necessary. After hospital discharge three days postoperatively, the patients were seen for follow up on the fifth day. If no complications were recorded, the patients were followed up on the ninth (when suture was removed), sixteenth and thirtieth post-operative day, and each two months for six months, and at least every twelve months thereafter. In cases of any complication the patients were seen as needed.

Testicles were considered to be abnormal if they were seen per-surgically to have both of the following characteristics: (1) loss of the typical ovoid shape and noticeable volume reduction by inspection and (2) thickness of vaginal visceral and albuginea layers, both by inspection and palpation (increased consistency). During follow-up, reappearance of intra-scrotal fluid of any volume suspected by physical examination and confirmed by ultrasound was defined as hydrocele recurrence. Recurrence was also considered to be present if intrascrotal fluid was detected only by ultrasound.

### Statistical Analysis

A two-tailed binomial test was used to compare proportions with a hypothesized value. Differences in proportions were tested using Fisher's exact test or Pearson's chi-square test. Differences in means were tested using a two-tailed Student's t test.

## Results

All patients from Group 1 and Group 2 were operated on between March 1994 and March 2001. No patients dropped out. It was not possible to define the duration of disease. Patients gave different answers when asked on several occasions during the study and their responses did not refer to the onset of the disease, but to the time when the volume of the hydrocele began to bother them. Thus, the duration of the disease could not be obtained in a reliable manner and is not presented. Only patients from Group 2 presented with more than one hydrocele sac. Orchiectomy was not performed on any of the patients included in the present study. Oral analgesic was needed only in 31 patients within 24 hours postoperatively (30 in Group 1 and one in Group 2). There was no postoperative haematoma or infection. Chronic edema, elephantiasis or lymphscrotum were not seen in the scrotal wall in any patients during the follow-up period (from five to 12 years).

### Group 1

General information about the patients, the characteristics of their hydroceles, hydrocelectomy and recurrence are found in Table 1. Two of the 968 patients experienced recurrence. One of them recurred twice (case 1). They were re-operated and the findings are described below.

**Table 1:** Characteristics of Group 1 patients who underwent hydrocelectomy at NEPAF.

		p
No. of men with hydrocele	968	
No. (%) bilateral	174*/968 (17.8%)	
No. (%) unilateral	794/968 (82.%)	
No. (%) on right	411/794 (51.7%)	0.3†
No. (%) on left	383/794 (48.2%)	
Mean (range) age (years) at the time of hydrocelectomy	30.33 (18–40)	
Total no. of hydroceles	1,142	
Total no. of hydrocele with straw colored fluid	1,128*	
No. (%) of patients with hydrocele recurrence	2/968 (0.2%)	
No. (%) of recurrent hydroceles	3**/1,128 (0.3%)	

*In 14 of 174 patients with bilateral hydrocele the fluid in tunica vaginalis was straw colored aspect on one side and milky on the contra lateral side. The milky side was excluded from the present study.

**One patient recurred twice.

**†:** Binomial test: hypothesized value = 50%.

#### Case 1

At first hydrocelectomy a slightly thick-walled right hydrocele containing dilated lymphatic vessel in a 25 year-old patient was completely excised after evacuating 168 mL of straw-colored fluid. At the fifth postoperative day, physical examination revealed a mild scrotal wall edema. The right scrotal content was slightly more adherent to the scrotal wall than was normally seen in other patients of this series. The patient was then seen more often. At the 3-month follow up examination a small quantity of fluid around the testis was perceived by physical examination and confirmed by ultrasound. The scrotal wall did not present any edema. During subsequent examinations the amount of fluid increased slowly. The recurrent hydrocele sac or simply the recurrent sac located on the anterior surface of the testis was excised six months after the first procedure. The gross appearance of the sac wall and the fluid (86 mL) were similar to the first hydrocelectomy.

A right hydrocele recurred for the second time two months after the second surgical procedure. The patient was operated on four months later. During this third operation, a small area located just close to the tail of the epididymis could be identified, from which a small quantity of a clear and slightly yellow fluid dripped slowly and continuously. It was likely generated by a ruptured lymphatic vessel not identified in prior surgeries. The recurrent sac, again slightly thick-walled, was excised. The leaking point was sutured with fine chromic catgut. Throughout his subsequent 11 years of follow-up, he was symptom-free and no hydrocele recurrence was detected, either both by physical or ultrasound examination.

#### Case 2

At first hydrocelectomy a thin-walled right hydrocele sac was excised in a 38 year-old patient with 216 mL of fluid. Five months later in a follow up visit, inspite of the patient being symptom-free, a small rounded cystic collection was palpable above the upper pole of the testis which was anechoic by ultrasound. Three months later the patient was re-operated, excising completely an isolated small thin-walled cystic hydrocele located on the anterior aspect of the cord, right above the upper pole of the testicle. The sac contained nine mL of pale straw-colored fluid. The patient was followed up for ten years afterward with no hydrocele recurrence, both by physical and ultrasound examination.

In these both recurrent cases the testicles appeared normal in size, shape and consistency.

### Comparison Group

Of the 436 patients presenting at NEPAF in whom previous hydrocelectomy was performed in another hospital, 93 presented hydrocele recurrence (21.3%). It was possible to obtain the information about the surgical technique used to repair the hydroceles in 218. Characteristics of the CG are found in Table 2. In patients for whom the medical records could not be retrieved, the hydrocele recurrent rate was 23.4%. No abnormalities in the scrotal wall such as chronic edema, elephantiasis or lymphscrotum, were seen in any of the 436 patients at physical examination.

**Table 2:** Characteristics of Comparison Group patients who underwent hydrocelectomy outside of NEPAF.

		p
No. of men	218	
No. (%) bilateral	24/218 (11.0%)	
No. (%) unilateral	194/218 (89.0%)	
No. (%) on right	98/194 (50.5%)	0.9*
No. (%) on left	96/194 (49.5%)	
Mean (range) age (years) at the time of hydrocelectomy	31.54 (19–40)	
Total no. of initial hydrocelectomies	242	
Surgical technique at previous surgery		
No. (%) eversion with partial excision	126/242 (52.1%)	
No. (%) eversion without partial excision	116/242 (47.9%)	
No. (%) of patients with hydrocele recurrence	42/218 (19.2%)	
No. (%) bilateral	02/42 (47.6%)**	
No. (%) unilateral	40/42 (95.2%)	
No. (%) on right	18/40 (45%)	
No. (%) on left	22/40 (55%)	
Surgical technique at previous surgery in patients with recurrence		0.3†
No. (%) eversion with partial excision	19/42 (45.2%)	
No. (%) eversion without partial excision	23/42 (54.7%)	

*Binomial test: hypothesized value = 50%.

**There was recurrence in only one side. In both cases the recurrent hydrocele was on the right side.

**†:** Pearson Chi-square test.

### Group 2

**Figure 1:**
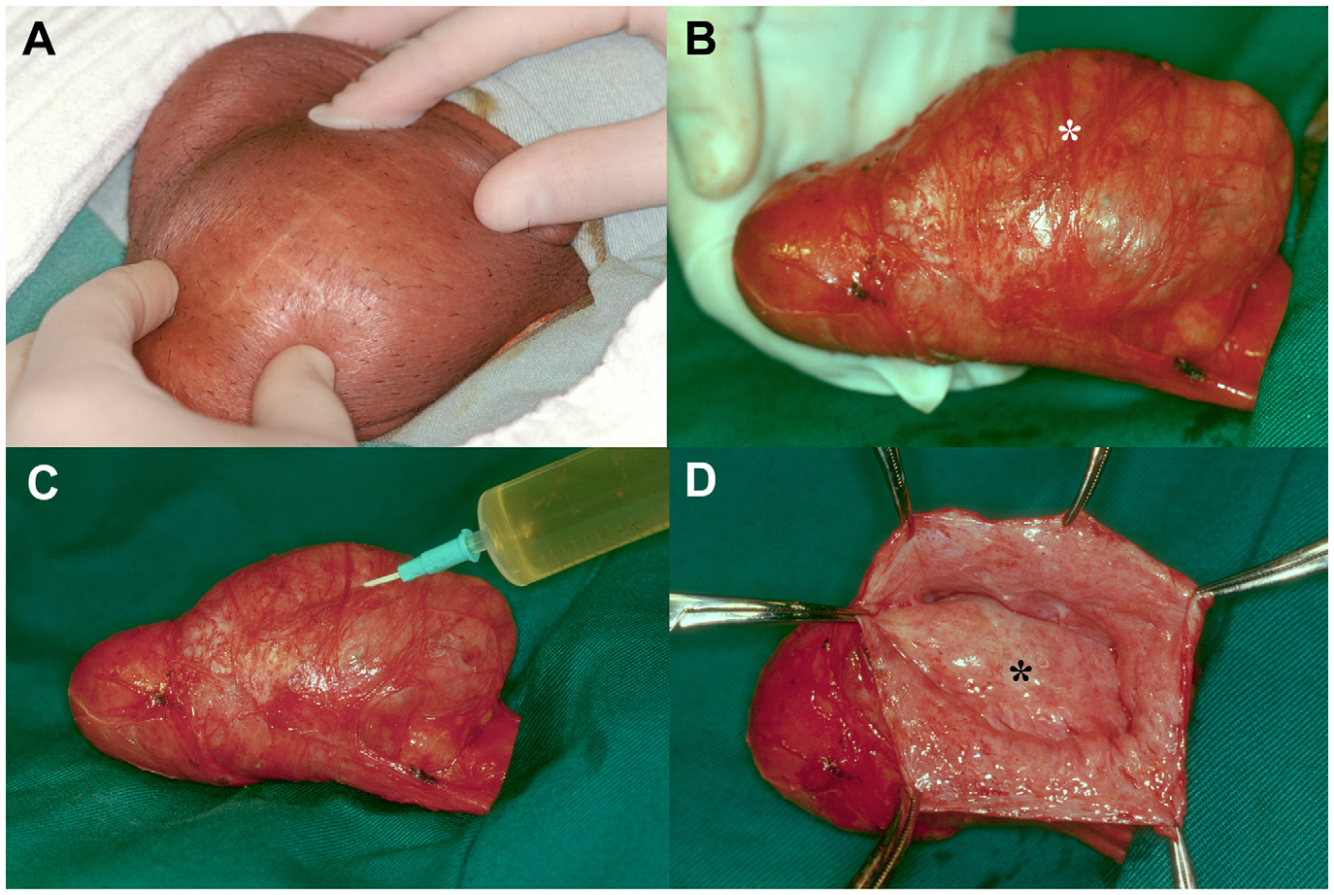
Anterior recurrence. Left recurrent hydrocele in a 31 year-old patient from G2. (A) Previous hydrocelectomy scar. (B) Recurrent hydrocele sac situated anterior to the testis (asterisk). (C) Aspiration of clear, straw colored fluid from anterior recurrent sac. (D) Opened recurrent hydrocele sac showing the anterior surface of the testis covered by tunica albuginea and visceral layer of tunica vaginalis (asterisk). By gross appearance the testicle is abnormal in shape and size. The sac wall (forceps) and testicular tunicas appear thick and with irregular surfaces.

**Figure 2:**
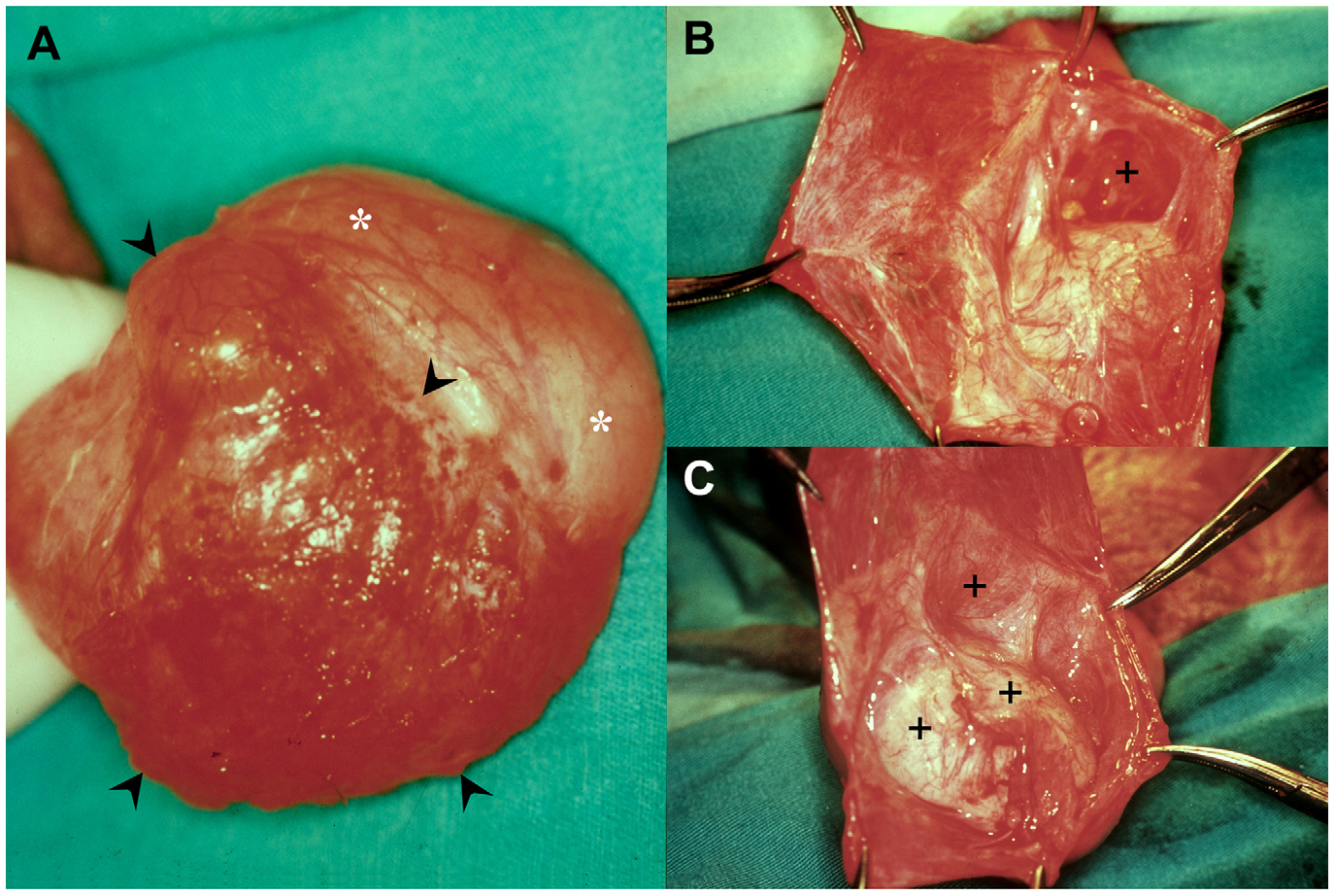
Posterior recurrence. Left recurrent hydrocele in a 36 year-old patient from G2. (A) Posterior recurrent hydrocele sac (arrow heads) and anterior surface of the testis (asterisks); (B) and (C) different views of opened posterior recurrent hydrocele sac showing multiple cavities and irregularity of its inner surface (**+**).

**Figure 3:**
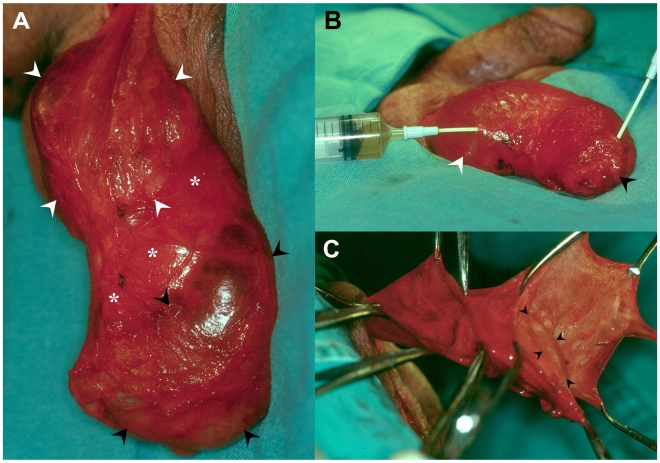
Mixed recurrence. Right recurrent hydrocele in a 29 year-old patient from G2. (A) Recurrent hydrocele sacs situated anterior (black arrow heads) and posterior (white arrow heads) to the testis (asterisks). (B) Emptied anterior recurrent hydrocele sac (black arrow head); aspiration of posterior recurrent hydrocele sac (white arrow head). (C) Opened, anterior (right forceps) and posterior (left forceps) recurrent hydrocele sacs; anterior surface of abnormal testis (arrow heads) is seen.

Of the 42/218 patients with recurrence and medical records retrieved, 28 agreed to a second operation at NEPAF. The surgical technique used previously was eversion with and without partial excision of the sac in 12 (42.8%) and 16 (57.2%) patients, respectively. The surgical approach for recurrent cases was the same used in G1. The twenty eight unilateral cases in G2 could be classified in three categories according to the location of the recurrent sac, as seen at surgery: (1) anterior recurrence: In five patients (17,8%) the recurrent sac was covering the anterior surface of the testis (Figure 1); (2) posterior recurrence. In eleven patients (39,3%) the returning sac was located on the posterior aspect of the testis and the epididymis (Figure 2) and (3) mixed recurrence. Twelve patients (42,8%) presented at least two separate recurrent sacs. They were located anterior and posterior to the testicular surface (Figure 3).

**Figure 4:**
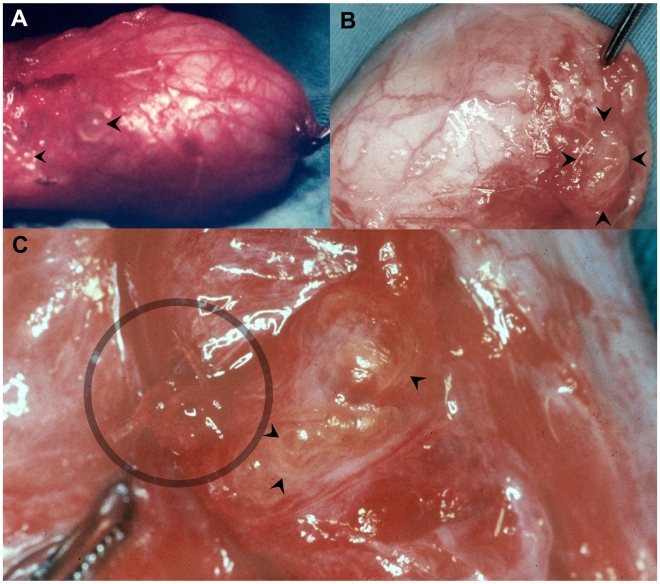
Lymphangiectasias and *W. bancrofti* adult worms seen through the lymph vessel wall. (A) A right emptied hydrocele sac in a 23 year-old patient from G1. Round dilations of lymphatic vessel (arrow heads) on the outer surface of the hydrocele sac wall. (B) A left hydrocele case in a 25 year-old patient from G1 after excision of hydrocele sac. Lymphangiectasia (arrow heads) in retrotesticular area, close to the excision margin of the hydrocele sac. (C) A closer view of B showing a cluster of small lymphangiectasias (circle). By transparency, filiform bodies of living worms (arrow heads) are seen in a larger lymphangiectasia.

During surgery in G1 and G2, in patients with slight or mild fibrosis in hydrocele sac and surrounding tissue, intact dilated lymphatic vessels and/or a small quantity of slowly dripping clear and slightly yellow fluid, could often be identified (Table 3). These lymphatic vessels and/or dripping process were seen in the inner or outer surface of the sac wall or in the retrotesticular area, near what became the resection-line when removing the sac (Figure 4).

**Table 3:** Comparison of age, lymphangiectasia, testicular abnormality findings, hydrocele volume, and hydrocele recurrence in Group-1, Control Group and Group-2 patients.

	GROUP 1	COMPARISON GROUP	GROUP 2	P
Mean (sd) age in yr at the time of the first hydrocelectomy	30.3 (6.2)	31.6 (5.5)	25.1 (4.3)	
Surgical findings:				
Lymphangiectasia	349/1,128 (30.9%)	NA	13/28 (46.43%)	0.081*
No. (%) on right	184/349 (52.7%)		06/13 (46.15%)	
No. (%) on left	165/348 (47.3%)		07/13 (53.85%)	
Testicle abnormalities	203/1,128 (18%)	NA	10/28 (35.7%)	0.025*
Mean hydrocele volume/mL** (range)	301 (72–1502)	NA	122.7 (58–396)	<0.001†
No. of recurrent hydrocele	3/1,128 (0.3%)	42/218 (19.3%)	0/28 (0%)	<0.001***
Mean (range) postoperative follow-up (mo)	103.78 (64–144)	NA	72.18 (60–83)	

*Fisher's exact test comparing groups 1 and 2.

**Geometric mean.

**†:** t-test.

***Fisher's exact test comparing group 1 and comparison group.

Marked changes in shape, volume, and consistency of the testis due to inflammatory reactions associated with fluid collection around the testis in G1 and G2 were seen significantly more so in G2 than G1 (p = 0,025). Testes seen at surgery to be abnormal, were affected with a range of severity (Table 3, Figure 1D and Figure 3C). The hydrocele volume was significantly lower in G2 patients (p = <0,001).

## Discussion

Hydrocelectomy dates from remote antiquity. The procedures employed have been devised and modified over the years resulting in a multiplicity of techniques with many variations and modifications of the original methods. It is generally believed that the pathogenesis of acquired hydroceles, irrespective of the etiology, is an unbalanced process between transudate production and reabsorptive activity of the tunica vaginalis parietal lymphatics [17], [18]. Thus, the rationale for open hydrocelectomies is to expose, permanently, the hydrocele fluid to an absorbing surface [19]. The mechanism of generating hydrocele has been considered to be the same in all acquired hydroceles and the etiology, per se, has not been considered when choosing the surgical approach. Fundamental factors influencing the choice of surgical procedure are efficacy, simplicity, safety, and, cost-effectiveness of the treatment. In addition to the surgeon's preference, the size of the hydrocele and the thickness of its sac wall have been considered elements that could be taken into account when deciding which technique to choose [19]–[24].

The hydrocele recurrence rates vary among studies [25]–[26] using different sample sizes, different criteria for recurrence, the type of the study (prospective or retrospective), degree of thickness of the hydrocele sac, the chosen surgical technique, different inclusion and exclusion criteria, different backgrounds of the health personnel involved and follow up periods. As a consequence, it is not easy to make accurate comparisons across studies.

The very significant difference in hydrocele rate recurrence (p<0,001) observed between patients from G1 and CG after two different surgical approaches – (1) complete excision and (2) eversion with or without partial excision of sac – could reflect more than differences in surgical technique. It may also signify that the pathogenesis of chronic filarial hydrocele is rather complex. The intrascrotal lymphatic vessels appear to be the preferred site for the adult worms of *W. bancrofti* in infected men. Extensive clinical, surgical and histological observations indicate that in almost 90% of infected men, adult *W. bancrofti* can be detected in the lymphatic vessels of the scrotal area [9], [10]. The primary lesion of bancroftian filariasis, while adult worms are alive, is non obstructive lymphatic vessel dilation without inflammation [8], [11]–[13]. Norões et al. [7] demonstrated that, by contrast, acute filarial hydrocele is a consequence of acute interruption of lymph flow from the tunica vaginalis of the testis. This obstruction is caused by filarial granuloma (corresponding to formation of palpable nodules detected by physical examination of intrascrotal contents [15]) resulting from death of *W. bancrofti* adult worms in the lumen of intrascrotal lymphatic vessels. They observed that 22% of patients who experienced nodule formation also developed acute hydroceles, and reabsorption of the granuloma led to resolution of 76% of such acute hydroceles within seven months during an eighteen month follow up period. Risk of acute hydrocele development, following a single filarial granuloma “event”, was increased by (1) the presence of nodules located in the superior paratesticular area (or adjacent to the posterior part of the upper pole of the testicle), a critical site where the lymphatic drainage of the tunica vaginalis, epididymis and testis converges [27] and (2) the occurrence of multiple filarial nodules. However, the risk factors and mechanism that lead acute filarial hydrocele to persist and to progress toward a chronic condition are not completely understood. Norões et al. [7] speculate that factors such as adult worm burden, formation of additional nodules, the speed of the granulomatous recanalization process, and the degree of pre-existing sub-clinical lymphatic disfunction could contribute to the chronicity of the process. The low progression rate of this “obstructive” hydrocele stands in contrast to the high prevalence of chronic hydrocele in filariasis endemic areas.

Based on observations after operating on patients with chronic hydrocele from non-endemic and endemic areas, and comparing surgical findings, we believe the accumulation of fluid in the vaginal cavity of the testis, in a large proportion of chronic filarial hydrocele cases, may be due to a different pathogenetic mechanism.

The surgical findings in recurrent and non recurrent hydroceles in G2 and G1 respectively, support the conclusion that lymph fluid composes the hydrocele fluid, based on the following evidences: (1) per-operatively, intact dilated lymphatics and/or a small quantity of slowly dripping clear and slightly yellow fluid were continuously seen in approximately one-third of the patients in G1 and G2. When a dilated lymphatic vessel ruptures, it is very difficult to visualize the vessel itself, but with a careful examination it is possible to see the clear fluid flowing from the lymphatic fistula. On the other hand, the lack of leak-prone dilated lymphatic vessels and lymphatic fistula in patients with a thicker tunica vaginalis and albuginea can be explained, in principle, by the important inflammatory reaction triggered by lymph fluid leading to fibrosis; (2) in 82,1% (22/28 ) patients from posterior recurrence of the hydrocele recurrence cases (G2) where the fluid collection was in a cavity located behind the testis and epididymis, the cavities were formed by the everted sacs where the fluid collection was in direct contact with the outer surface of the everted hydrocele sac. As well known, this outer surface is not a serous lined layer which is not capable of producing fluid. In everted sacs, the lined serous layer was in direct contact with the scrotal wall where lymphatics are able to drain fluid production. This evidence argues against primacy for the concept that transudate production/reabsorption problems represent the essence of the majority of chronic hydrocele fluid accumulation in lymphatic filariasis.

Based on results of the current study, it seems reasonable to propose that, first, the straw colored “filarial hydrocele fluid” consists of a combination of clear lymph fluid and transudate, in various proportions. The major mechanism of chronic filarial hydrocele proposed in the present study, is the same for milky fluid accumulation in cases of chylocele. In chyloceles the difference is the presence of chylomicrons caused by diffuse and extensive high retroperitoneal lymphangiectasia promoting the retrograde flow of milky lymph in ruptured intrascrotal lymphatics (J. Norões, personal communication). Second, based on G2 findings the high hydrocele recurrence rate in CG was likely to be due to the presence of lymphatic fistula in everted hydrocele sac and/or in surrounding tissue.

Based on the current findings it is suggested that the term “filaricele” be introduced for chronic straw colored fluid accumulation in the vaginal cavity in endemic areas. This term would help to emphasize the differences in pathogenesis and in the recommended surgical techniques that are appropriate for acquired chronic filarial and non-filarial hydroceles. On the other hand, the term chylocele should be kept for the milky appearance of hydrocele fluid rich in chylomicrons.

Two other practical lessons were also learned from this study: (1) in the second recurrent case from G1, the sac extended up to the spermatic cord, and a small posterior segment of the sac wall, adherent to the cord, was inadvertently not excised at the first hydrocelectomy. During the re-operation a small portion of tunica vaginalis in the inferior part of the cord was found to contain a small cystic hydrocele. Thus, excision of the tunica vaginalis should be as complete as possible; (2) in hydrocele among patients from non-endemic areas, the patient's personal preference as to whether or not he desires treatment should be given strong consideration since the indication for treatment depends, particularly, upon how much the hydrocele bothers him. By contrast, chronic filarial hydrocele could threaten the integrity of the testis even in small volume cases as shown in this study. Thus the medical indication for surgical treatment is stronger and the patient should be advised accordingly.

It was beyond of the scope of this study to compare the volume of all hydrocele cases, echogenicity of the fluid, spermogram profiles and microfilaraemia status, which are planned to be published separately.

One potential limitation of the study was the small sample size of Group 2. In spite of that, this group provided unprecedented detailed findings leading to a pioneering classification of recurrent hydrocele in endemic area occurring after eversion technique with and without partial excision of the hydrocele sac.

In conclusion, in bancroftian filariasis endemic areas, lymphatic fistulae are likely to be an important mechanism responsible for chronic hydrocele, whether recurrent or not. Particularly with the intent to avoid hydrocele recurrence and testicular damage, complete excision of the hydrocele sac with its dilated lymphatic vessels and/or lymphatic fistula is advised as is identification and suturing or excision of any visible dilated lymphatic vessels in surrounding tissue, whether leaking or not.
